# Evidence of on-going transmission of Shiga toxin-producing *Escherichia coli* O157:H7 following a foodborne outbreak

**DOI:** 10.1017/S0950268821001278

**Published:** 2021-06-07

**Authors:** Saira Butt, Alison Smith-Palmer, Allan Shand, Eisin McDonald, Lesley Allison, Jane Maund, Anand Fernandes, Bhavita Vishram, David R. Greig, Claire Jenkins, Richard Elson

**Affiliations:** 1 National Infection Service, Public Health England, 61 Colindale Avenue, London, NW9 5EQ, UK; 2 Health Protection Scotland, Glasgow, Scotland; 3 Food Standards Scotland, Aberdeen, Scotland; 4 Scottish E. coli O157/STEC Reference Laboratory, Edinburgh, Scotland; 5 South East PHE Centre, Public Health England, Hampshire, UK

**Keywords:** Food-borne zoonoses, molecular epidemiology, public health microbiology, Shiga-like toxin-producing *E. coli*, surveillance

## Abstract

In August 2019, public health surveillance systems in Scotland and England identified seven, geographically dispersed cases infected with the same strain (defined as isolates that fell within the same five single nucleotide polymorphism single linage cluster) of Shiga toxin-producing *Escherichia coli* O157:H7. Epidemiological analysis of enhanced surveillance questionnaire data identified handling raw beef and shopping from the same national retailer (retailer A) as the common exposure. Concurrently, a microbiological survey of minced beef at retail identified the same strain in a sample of minced beef sold by retailer A, providing microbiological evidence of the link. Between September and November 2019, a further four primary and two secondary cases infected with the same strain were identified; two cases developed haemolytic uraemic syndrome. None of the four primary cases reported consumption of beef from retailer A and the transmission route of these subsequent cases was not identified, although all four primary cases visited the same petting farm. Generally, outbreaks of STEC O157:H7 in the UK appear to be distinct, short-lived events; however, on-going transmission linked to contaminated food, animals or environmental exposures and person-to-person contact do occur. Although outbreaks of STEC caused by contaminated fresh produce are increasingly common, undercooked meat products remain a risk of infection.

## Introduction

Shiga toxin-producing *Escherichia coli* (STEC) O157:H7 emerged as a gastrointestinal pathogen of public health concern in the early 1980s [1]. Compared to other bacterial pathogens, infection is rare, but symptoms are severe, including bloody diarrhoea, abdominal pain, vomiting and fever [2]. In England, over one-third of cases are hospitalised, and a subset of patients develop haemolytic uraemic syndrome (HUS) characterised by renal dysfunction, and/or cardiac and neurological complications, that can be fatal [2, 3].

Transmission to humans occurs via the consumption of contaminated food, direct contact with colonised animals or their environment. Foodborne outbreaks of STEC O157:H7 in England have been associated with contaminated raw or undercooked meat, or cooked meats which had been cross-contaminated; raw milk and raw milk products and contaminated raw vegetables and salads [4]. The infectious dose is low and there is evidence of person-to-person transmission in households and institutional settings [2].

Whole genome sequencing (WGS) provides highly discriminatory typing for public health surveillance, outbreak detection and investigation. Prior to the implementation of WGS in the UK, the relatedness of isolates of STEC O157:H7 epidemiologically linked to the same outbreak was evaluated [5, 6]. The analysis showed that isolates from cases epidemiologically linked to the same outbreak fell within the same five single nucleotide polymorphisms (SNP) single linkage cluster. In light of these findings, routine surveillance algorithms involve the review of epidemiological data linked to cases of STEC O157:H7 infected with isolates falling within this threshold to look for common exposures. This approach has led to the resolution of outbreaks of STEC O157:H7 caused by a wide range of contaminated food vehicles, and common animal/environmental exposures, mostly associated with relatively large temporally related clusters of cases [4]. Where the number of outbreak cases is small, and/or geographically or temporally dispersed, a common exposure may be difficult to confirm, even when the SNP typing indicates the cases are infected with the same strain [6].

Public health institutions in England and Scotland operate enhanced surveillance systems for STEC across the UK [2, 7]. For every laboratory-confirmed case of STEC O157:H7, a detailed history is obtained for the 7 days prior to onset of illness using an enhanced surveillance questionnaire (ESQ), and isolates of STEC O157:H7 linked to each confirmed case are genome sequenced [6, 7]. In August 2019, joint surveillance activities in England and Scotland identified an outbreak of STEC O157:H7 phage type (PT) 21/28. Before and after the outbreak was identified, routine surveillance identified additional isolates that fell within the same five SNP cluster as the outbreak strain but were temporally distinct. In this study, we describe the co-ordination of the multi-agency investigation of the outbreak, the key findings from analysis of the shared epidemiological and microbiological enhanced surveillance data, and the post-outbreak investigation of the phylogenetically related but temporally distinct cases.

## Methods

### Clinical microbiology

In the UK, faecal specimens from all patients submitted to local hospital microbiology laboratories are tested for the presence of *Salmonella*, *Campylobacter*, *Shigella* spp. and STEC O157:H7. Presumptive isolates of STEC O157:H7 were sent to the PHE Gastrointestinal Bacteria Reference Unit (GBRU) or Scottish *E. coli* O157/STEC Reference Laboratory (SERL) for confirmation, identification of PT and the presence of Shiga toxin (*stx*) genes by PCR. WGS was undertaken as described previously [7, 8].

### Outbreak detection

All faecal specimens in the UK are tested for STEC O157:H7 and isolates from local hospital laboratories in England, Wales and Northern Ireland are referred to Public Health England (PHE) for confirmation and typing using WGS of all STEC. In Scotland, isolates are referred to SERL, and WGS data are shared between the two reference laboratories to monitor for cross-border outbreaks.

### Epidemiological investigation

PHE convened a multi-disciplinary Incident Management Team (IMT) with membership drawn from Public Health Scotland, the Food Standards Agency (FSA) and Food Standards Scotland (FSS). The aims of the IMT were to investigate the outbreak and identify potential food vehicles, routes of transmission and implement appropriate control measures. The objectives of the epidemiological investigation were to identify and describe cases associated with the outbreak, and to identify and confirm the likely source/vehicle and make recommendations for control measures.

The IMT agreed the following case definitions:

Confirmed: A case of STEC O157 PT 21/28 confirmed by GBRU belonging to the five-SNP designation 4.4.4.955.4673.5005.%.
Probable: A case of STEC O157 PT 21/28 reported by GBRU or SERL and awaiting sequencing.

Prospective and retrospective case ascertainment was undertaken by reviewing PT and WGS data for STEC cases reported in 2019. Historical sequencing data from food, environmental and animal samples were compared to the sequence profiles of outbreak cases. To determine whether there were cases further afield, the UK posted details of the outbreak and WGS accession numbers on the Epidemic Intelligence Information System, a web-based international notifications and communications platform managed by the European Centre for Disease Control and Prevention (https://www.ecdc.europa.eu/en/publications-data/epidemic-intelligence-information-system-epis).

### Hypothesis generation

Details of clinical symptoms, travel history and exposure to food, water and animals reported by cases preceding their illness were extracted from enhanced surveillance systems in England and Scotland. Cases were re-interviewed using a comprehensive trawling questionnaire to capture more detailed information on exposures of interest identified via the enhanced surveillance systems. Interviews were conducted by a small number of trained people and a script was provided to ensure consistency.

### Analytical epidemiology

Despite small numbers of cases, a case-case study was performed. Data were extracted from the national enhanced surveillance system for the confirmed outbreak cases residing in England, and exposure data were provided by Health Protection Scotland for the confirmed cases residing in Scotland. Because of differences in the way that surveillance data are collected in Scotland and England, a bespoke dataset was created by combining standardised data collected from ESQs and the trawling questionnaires.

A ‘control’ was defined as a primary, symptomatic case of STEC O157 with an onset date between 1 September 2018 and 1 September 2019 and with a completed ESQ. This timeframe was selected to overlap with the outbreak cases' exposure periods and to cover a full year, as the descriptive epidemiology of the outbreak did not suggest a seasonal product. Cases were excluded if they had travelled outside the UK in the 7 days preceding their illness onset or if they were associated with a known outbreak.

#### Univariable analysis

Odds ratios were calculated for each food exposure reported by at least 50% of cases in the bespoke dataset, comparing the odds of outbreak cases reporting the exposure with the odds of comparison cases reporting the same exposure. The Fisher's exact test was conducted to produce *P*-values, to account for the small numbers of cases (<5 exposed).

#### Multivariable analysis

Variables where *P* < 0.20 and proportion exposed was >20% following univariate analysis were included in a multivariable analysis in addition to the *a priori* variables adulthood (>16 years) and sex. The Spearman correlation coefficient was calculated between each of the exposure variables in the full model to evaluate any potential collinearity. The final model was selected using backwards elimination, successvely dropping variables where *P* > 0.05 in a likelihood ratio test comparing the model with ans without the variable.

### Food and animal distribution networks

Food chain investigations were conducted by the FSA and FSS. Informed by the descriptive epidemiology and microbiology, this exercise aimed to trace the supply and distribution of potentially implicated products from their source to the point of sale or consumption.

Data from supermarket loyalty card schemes were requested from retailers in order to identify food exposures common to cases during the 4 weeks prior to onset of illness, in order to capture products with longer shelf lives such as frozen foods or foods that could be frozen after purchase. Data on the movement of cattle during the relevant time period were requested from the Animal Reporting & Movement Service (ARAMS) (http://www.arams.co.uk/), a system which records the movements of cattle, sheep, goat and deer between agricultural premises, livestock markets and slaughterhouses in the UK.

### Environmental investigations including sampling

Environmental investigations conducted at premises linked to food products or animal exposures included reviews of routine samples taken as part of process hygiene criteria at approved premises, assessment of the management of animal-based attractions, collection of veterinary samples and sampling of the wider environment using boot-socks (a previously described method of sample collection originally conceived to detect *Campylobacter* present in the environment http://enigmaproject.org.uk/the-project/) (online Supplementary Table).

## Results

### Descriptive epidemiology of the outbreak

**Fig. 1.**
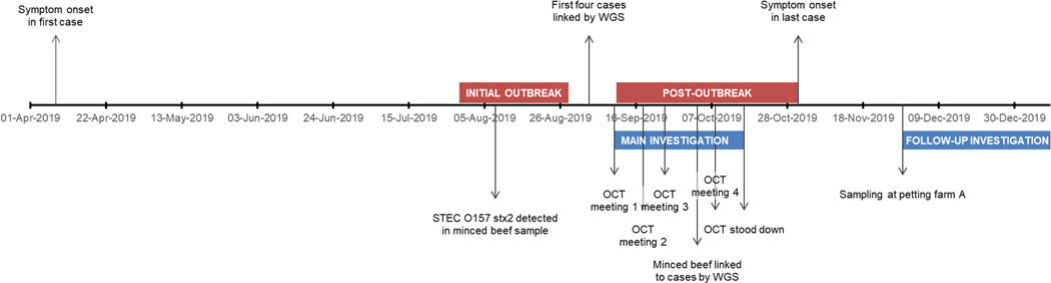
Timeline of key events in the investigation.

**Fig. 2.**
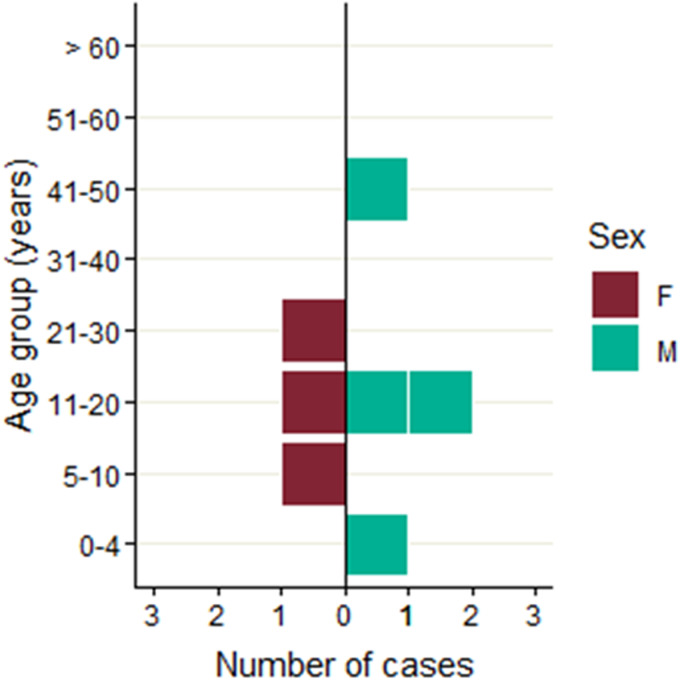
Age-sex distribution of confirmed cases in initial outbreak (*n* = 7).

**Fig. 3.**
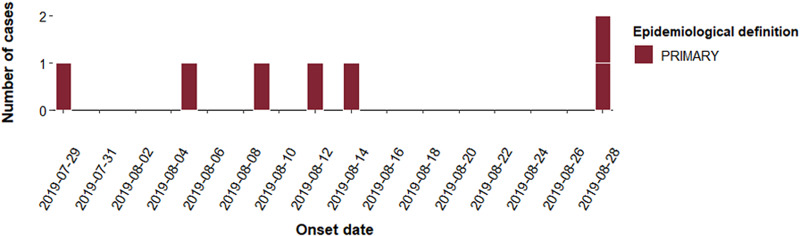
Epidemic curve of confirmed cases in initial outbreak by onset date.

**Fig. 4.**
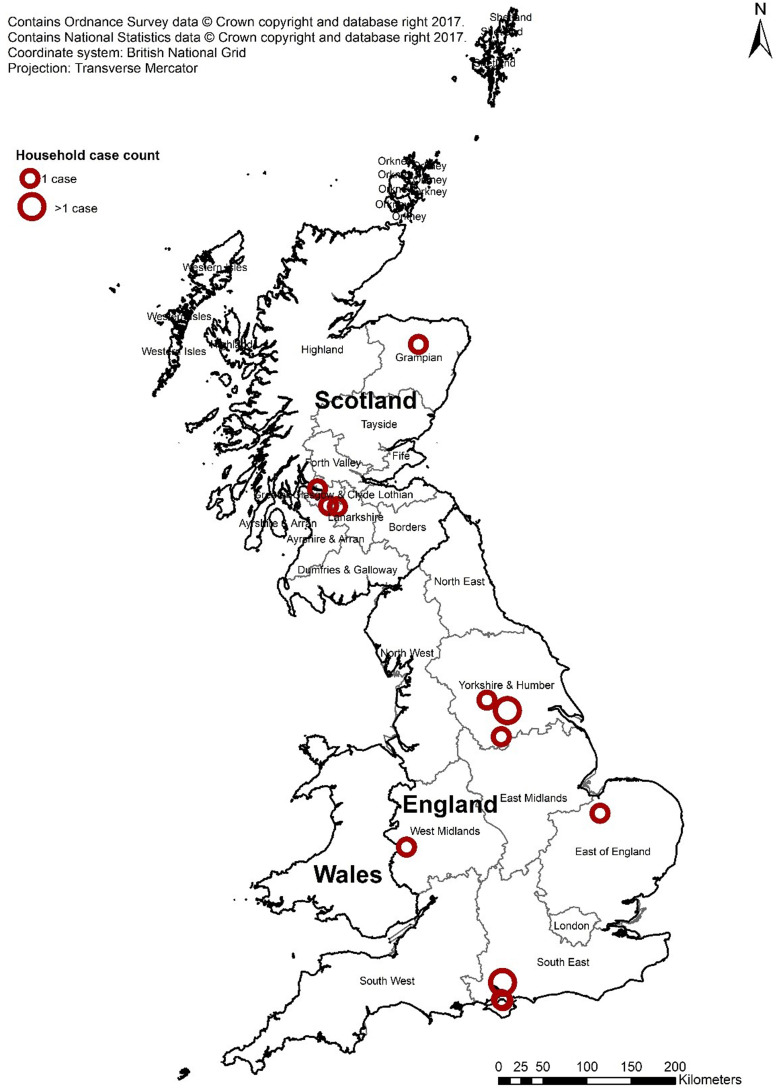
Geographical distribution of all confirmed cases in the five-SNP cluster. There are two one-case households in the South East of England that are indistinguishable due to proximity.

In August and September 2019, seven cases of STEC O157:H7 with an identical SNP profile were identified through routine surveillance (Fig. 1). Four of the cases lived in England and three lived in Scotland. The majority of cases (4/7; 57%) were male and ages ranged from 1 to 46 years with the median age 19 years (Fig. 2). All seven cases were identified as primary cases. The earliest onset date was 29 July 2019 and the last was 28 August 2019 (Fig. 3). The residential locations of cases are shown in Figure 4. All seven cases reported diarrhoea or bloody diarrhoea, five were admitted to hospital, two reported fever, and none developed HUS. No deaths due to STEC O157:H7 infection were reported.

The causative pathogen was identified as STEC O157:H7 PT21/28 harbouring *stx* subtype *stx2a/stx2c*. This strain was unique and had not been detected in the preceding 3 years of routine WGS for STEC isolates in England and fell within the same clade as STEC O157:H7 isolated from UK beef cattle isolated during a surveillance study conducted in 2015 [9].

**Fig. 5.**
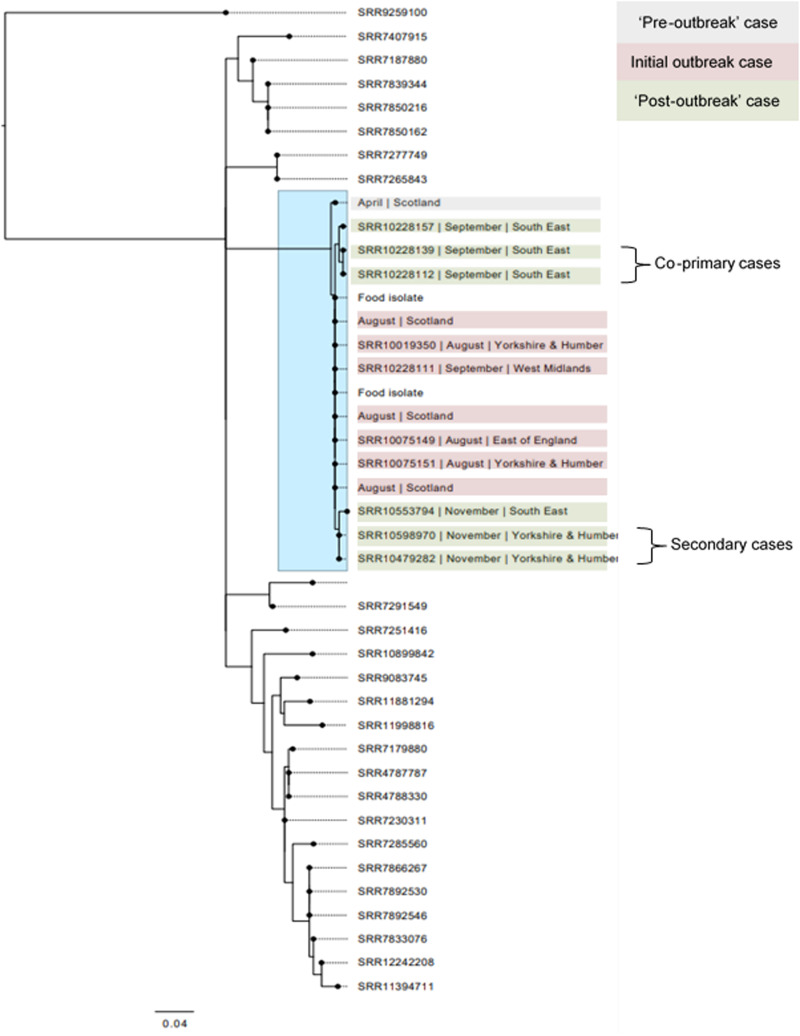
Phylogenetic relationship of the isolates linked to the outbreak and the strains isolated pre- and post-outbreak. Isolates in the blue box all fall within the same five SNP single linkage cluster.

A retrospective review of surveillance data held at Public Health Scotland identified a further case whose isolate clustered within five SNPs of the outbreak strain (Fig. 5). The case from early April 2019 was an adult from the North of Scotland who reported contact with farm animals including cattle.

### Hypothesis generation

Six of the seven cases in July and August reported shopping at the same national retailer (retailer A) and five reported the consumption of minced meat, burgers or sliced ham from the delicatessen. Of these, two cases specifically reported handling raw minced beef or raw beef burgers. Trawling interviews were completed for four of the six cases that had been identified at that point in time to capture detailed information on exposures of interest identified from the routine surveillance questionnaires. All four reported consumption of food purchased from retailer A in the week before onset; three reported handling or consuming minced beef of whom two had purchased the minced beef from retailer A. At the first IMT meeting, FSS shared the results of a microbiological survey they were conducting examining the prevalence of pathogens and hygiene indicator organisms of minced beef on retail sale in Scotland (https://www.foodstandards.gov.scot/publications-and-research/scientists-and-researchers/food-safety-research). Two samples taken from the same branch of retailer A on 29 July 2019 were positive for STEC O157 stx2, detected on 08 August 2019. Although this was not a branch that any of the cases had reported shopping at, these were subsequently confirmed as the outbreak strains by WGS (Fig. 5).

### Analytical epidemiology

The seven cases identifed in July and August and 73 controls were included in the case-case study to test the hypothesis that exposure to raw beef and/or shopping at retailer A was associated with infection. Univariate analysis showed increased odds for shopping at retailer A (OR 33.82 (3.37–1578.85), *P* < 0.001) or handling raw beef (OR 10.83 (1.46–83.67), *P* = 0.008) among cases.

There was evidence for increased odds with handling raw poultry (OR 4.62 (0.82–41.62), *P* = 0.042) or consuming cooked pork (OR 4.80 (0.71–52.70), *P* = 0.096).

Strong collinearity was not identified between any of the variables, and so the following variables were included in the multivariable analysis along with age and sex: exposure to retailer A, handled raw beef, ate cooked pork and handled raw poultry. The final model included handling raw beef (OR 9.35 (0.93–94.11), *P* = 0.058) and exposure to retailer A (OR 30.63 (2.67–351.65), *P* = 0.006) in addition to age and sex, with the LRT *P* < 0.001 when compared to the null model. Adding interaction terms between handling raw beef and exposure to retailer A did not improve the performance of the model.

### Food chain investigations

Traceback of the food sample from retailer A that tested positive for STEC O157:H7 during the FSS microbiological survey of minced beef revealed that it was sourced from a Scottish cow, slaughtered in Scotland and minced at a cutting plant in England owned by retailer A. The minced beef from that cutting plant was predominately distributed via retailer A but other retailers were also supplied. Purchase data linked to retailer A loyalty cards were available for three of the six cases identified at that point in the investigation. During the 4 weeks prior to onset of symptoms, all three cases purchased raw beef products, although a common product was not identified.

### Investigation of on-going transmission of the outbreak strain

In late September 2019, colleagues from the South East PHE Centre alerted the national Gastrointestinal Infections Team of three cases of presumptive STEC O157:H7 who had visited petting farm A on the same day. Two cases were household contacts but were unknown to the third case. GBRU confirmed the isolates were STEC O157:H7 PT21/28 and part of the same five SNP cluster as the outbreak cases linked to minced beef (Fig. 5). In November 2019, an additional three cases (one primary and two secondary cases) belonging to the same five SNP cluster as the outbreak strain were identified (Fig. 5). The primary case in this additional group of three had visited petting farm A during their incubation period and subsequently travelled to visit relatives in the north of England, resulting in the two secondary cases. All four primary cases who visited the petting farm denied direct contact with the cattle. An overview of the investigation timeline is provided in Figure 1.

**Fig. 6.**
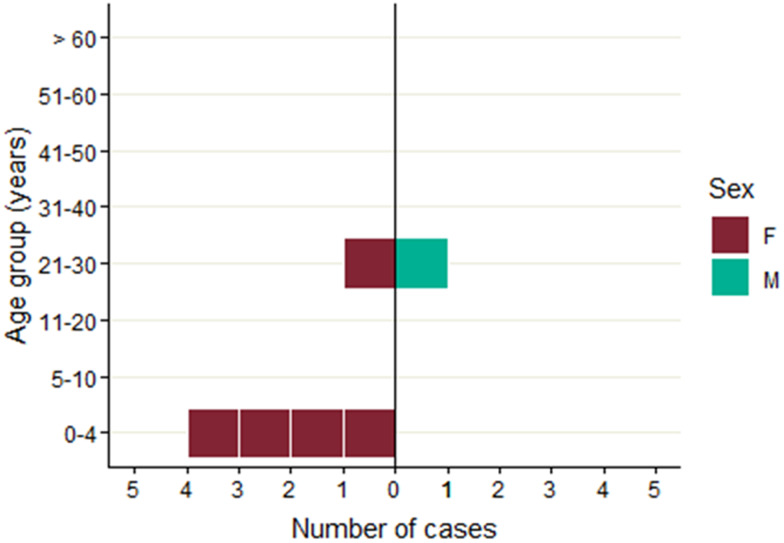
Age-sex distribution of post-outbreak cases (*n* = 6).

**Fig. 7.**
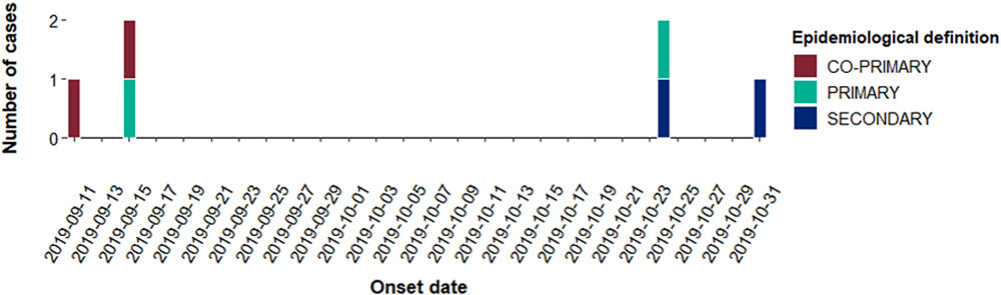
Epidemic curve of post-outbreak cases by epidemiological definition and onset date (*n* = 6). Onset of secondary case occurs on the same day as the linked primary case – reported onset was uncertain and ranged over several days, but was reported to be definitely after the primary case. The secondary case was defined as such based on this information and the primary case's epidemiological link to the petting farm which the secondary case did not have.

All six ‘post-outbreak’ cases lived in England. All but one case (5/6; 83%) were female and ages ranged from 2 to 30 years (Fig. 6), with the median age being 4 years. Three cases (50%) were admitted to hospital, two (34%) reported bloody diarrhoea, one (17%) reported fever, and two (33%) developed HUS – both of whom were children. No deaths due to infection were reported. The earliest onset date was 11 September 2019 and the last was 4 November 2019 (Fig. 7).

### Environmental investigations

Inspection by Environmental Health Officers (EHOs) of the premises at petting farm A concluded that the facilities and hygiene standards were satisfactory, with no major concerns. At the request of the IMT, 26 faecal samples from different species and at different locations across petting farm A were obtained for testing on 2 December 2019 (Fig. 1). All samples tested negative for STEC O157:H7. Boot sock samples from walking through the premises were also negative. Data on the movements of the animals that had been on the farm demonstrated no overlap with the source of the implicated beef. The movement data on sheep and cattle from petting farm A revealed no commonalities with the movement of the cattle identified from the matching minced beef isolate.

Only one of the ‘post-outbreak’ primary cases reported consumption of beef and none reported shopping at retailer A. None reported eating food provided at the petting farm, and only the co-primary cases from the same household reported dining at a common catered venue. Supply chain investigations for the petting farm catering as well as other catered venues visited by the ‘post-outbreak’ primary cases were conducted to identify if any were supplied by the implicated cutting plant. No commonalities with the distribution chain for the implicated minced beef were identified.

### Control measures

All cases and contacts were managed by PHE HPTs or NHS Scotland HPTs and local EHOs in line with recommendations in the national guidance (https://assets.publishing.service.gov.uk/government/uploads/system/uploads/attachment_data/file/732569/Interim_public_health_operational_guidance_for_STEC_PDF.pdf t; https://hpspubsrepo.blob.core.windows.net/hps-website/nss/2032/documents/1_SHPN-Guidance-Ecoli-shiga-STEC.pdf). This included the exclusion and screening of cases and contacts in risk groups to reduce the risk of further spread. Advice on hand and food hygiene was provided to all cases and contacts together with guidance on environmental cleaning and disinfection.

As minced beef is a non-ready to eat (RTE) product, legislation does not require these products to be free of pathogens, as the risk of infection is attenuated with adequate food handling and cooking practices. Additionally, as the prevalence of STEC in minced beef as calculated in the FSS minced beef survey was not above standard levels accepted across Europe (https://acmsf.food.gov.uk/sites/default/files/acm_1191_stec.pdf), the risk was determined to be within usual limits.

## Discussion

This report summarises the investigations and management of an outbreak and on-going transmission of a highly pathogenic strain of STEC O157 PT21/28 that occurred in England and Scotland in 2019. The IMT concluded that the source of infection was most likely Scottish cattle based on the fact that the isolates from humans and Scotch beef samples fell within a five-SNP cluster, the onsets of the majority of the cases followed the identification of the outbreak strain in minced beef in July 2019 and that Scotch beef is a protected geographical indication that animals were born and reared in Scotland.

The relative importance of different vehicles in causing outbreaks in England has changed over time [10]. Early outbreaks of STEC O157:H7 were caused by contaminated meat products, including minced beef and beef burgers [11]. Since significant meat hygiene practices were implemented in the late 1990s, outbreaks of STEC O157:H7 in England caused by meat products have been detected less frequently [10]. Conversely, detection and investigation of outbreaks associated with fresh produce, including salad vegetables, have increased in regularity [12–14]. However, outbreaks linked to meat products do still occur in the UK, including outbreaks linked to venison, beef burgers and cross-contamination of raw and cooked meat products on a butcher's premise [15–17]. Measures to prevent infection from contaminated food include adequate cooking of meat products before consumption and avoiding cross-contamination of RTE products from raw meat.

The SNP variation within the WGS cluster (including food isolates) was comparable to previous foodborne outbreaks linked to RTE and non-RTE foods, but only half (7/14) of the total number of cases linked to the five SNP cluster could be explained by exposure to raw beef products, sold via retailer A. There was no evidence that the remaining cases were infected by the same food vehicle. It is possible that the first infection in April 2019 may have been as a result of direct exposure to animals or environmental contamination based on the case's residential location. Alternatively, although the case did not report beef consumption prior to onset of symptoms, contaminated food as a possible exposure cannot be ruled out. On-farm sampling of cattle has proven useful for establishing farm to fork transmission during previous outbreak investigations [15, 18, 19]; however, information tracing the animals back to individual farms was not made available to the IMT.

As well as reporting different exposures, the six ‘post outbreak’ cases that fell within the same five SNP single linkage cluster were also temporally and spatially distinct from the outbreak cases. These subsequent cases were temporally dispersed but only one reported consumption of beef and none reported shopping at retailer A. All the primary and co-primary cases (the two cases in the same household) in the on-going transmission cluster reported exposure to petting farm A. Although animal movement data did not indicate any contact between the animals at petting farm A and the cattle from which the implicated minced beef came, it is possible that there was an indirect connection between the two. Furthermore, although the catering supply chain for the petting farm did not align with the established distribution chain of the implicated mince beef, alternative distribution chains may have been in operation.

The six cases identified after the initial outbreak cases appear to be the result of transient on-going transmission of the outbreak strain either via the food chain, contact with animals or via person-to-person contact. For the most part, outbreaks of STEC O157:H7 in the UK appear to be distinct, short-lived events; however, the phenomenon of transient on-going transmission has been observed previously in outbreaks linked to both contaminated food and environmental exposures [13, 15, 20]. On-going transmission of ‘outbreak’ strains of *Salmonella* species has also been observed [21, 22].

The majority of foodborne outbreaks of STEC O157:H7 in the UK are small and geographically dispersed, and it is often difficult to determine the vehicle of infection. In this investigation, we were able to identify the vehicle because of a co-incidental finding from microbiological surveillance of minced beef at retail. This finding was supported by the epidemiological analyses; however, it is unlikely that the epidemiological evidence alone would have been accepted as sufficient evidence. Despite identifying the vehicle and implementing public health measures to prevent on-going person-to-person transmission, subsequent cases were identified, including two cases of HUS. We were unable to ascertain the infection route of the primary cases that occurred after the initial outbreak cluster. We were unable to identify retailers other than retailer A that were supplied by the cutting plant linked to the outbreak cluster, or whether cattle from the same herd linked to the minced beef sample were sent to be slaughtered at a different abattoir and cutting plant supplying other retailers and food outlets. Tighter regulations and more transparency around food distribution chains would greatly enhance foodborne outbreak investigations such as this and may provide information to prevent on-going transmission. We also recommend focusing on sampling further back in the food chain to provide prevalence information for animals, water and the on-farm environment.

## Data Availability

FASTQ reads from all sequences in this study can be found at the PHE Pathogens BioProject at the National Center for Biotechnology Information (accession number: PRJNA315192).

## Supplementary material

For supplementary material accompanying this paper visit http://dx.doi.org/10.1017/S0950268821001278. click here to view supplementary material
